# An Energy-Efficient Slotted Sense Multiple Access Broadcast Protocol for Reliable Command Delivery in Dynamic Wireless Sensor Networks

**DOI:** 10.3390/s19051236

**Published:** 2019-03-11

**Authors:** Dae-Seung Yoo, Van Khoe Ta, Byung-Tae Jang, Hoon Oh

**Affiliations:** 1 Electronics and Telecommunications Research Institute, Daejon, Korea; ooseyds@etri.re.kr; 2 Department of Electrical and Computer Engineering, University of Ulsan, Ulsan, Korea; tavankhoe@gmail.com

**Keywords:** sharable slot, slotted broadcast, tree, node mobility, energy-efficient

## Abstract

In industrial monitoring and control applications, a server often has to send a command to a node or group of nodes in wireless sensor networks. Flooding achieves high reliability of message delivery by allowing nodes to take the redundancy of receiving the identical message multiple times. However, nodes consume much energy due to this redundancy and the long duty cycle. A reliable slotted broadcast protocol (RSBP) tackles this problem by allocating a distinct broadcast slot (BS) to every node using a tree topology. Not only does it remove collision, but it also minimizes energy consumption such that every node remains active only during its parent’s broadcast slot and its own broadcast slot to receive and rebroadcast a message, respectively. However, it suffers from low reliability in harsh environments due to the compete removal of redundancy and low responsiveness to the changes in network topology due to the global scheduling of slots. Our approach allocates one distinct broadcast sharable slot (BSS) to each tree level, thus making a BSS schedule topology-independent. Then, nodes at the same level compete to rebroadcast a message to nodes at one level higher within the BSS, thus allowing the redundancy. In addition, it uses a slot-scheduled transmission within BSS that can further improve reliability by reducing message collisions and also enables the precise management of energy. According to simulations and experiments, the proposed approach can achieve high reliability comparable to flooding and low-energy consumption comparable to RSBP.

## 1. Introduction

In harsh industrial fields, workers can be exposed to unexpected accidents. Damage can be reduced or avoided if the proper measures are taken in time. This requires a context-aware monitoring and control system in which a sink or server collects context data from sensor devices (nodes) installed in the target work field, judges the situation based on the analysis of the collected data, and warns the workers of any accident by sending a warning message to the nodes around the accident site. The slotted sense multiple access (SSMA) protocol deals with the problem of reliable data collection from sensor nodes in wireless sensor networks (WSNs) [1]. On the contrary, a sink or a server needs to send a message or command for warning to some nodes or actuators in the target field. In this case, the warning message has to be delivered in a reliable manner under a reasonable delay bound. However, it is difficult to achieve this due to time-varying link conditions caused by node mobility, and internal and external interferences (internal interference indicates an interference caused by different data transmissions in the same network, while external interference is caused by data transmissions in other wireless networks using the same frequency band) [2], and various obstructions such as steel materials, concrete blocks, etc. The energy management of a node is another critical issue due to the difficulty in replacing a battery. Thus, this paper aims at designing a protocol for a reliable and energy-efficient delivery of a command message in dynamic WSNs.

Various protocols for the transmission of a command message have been proposed so far and can be divided into three categories: flooding [3,4], selective rebroadcasting [5,6,7,8,9,10,11], and tree-based rebroadcasting [12,13,14]. The first category is a brute-force flooding approach in which a node rebroadcasts a received message after a random delay if it receives the message for the first time. It can achieve high reliability in message delivery; however, it causes high-energy consumption due to poor energy management, as well as the redundancy of transmitting and receiving or overhearing an identical broadcast message multiple times. The Glossy protocol [4] tried to reduce the flooding period with the use of capture effect, by which a node can demodulate a message successfully if it receives multiple identical messages within the time difference of 0.5 µs or smaller. Thus, every node rebroadcasts a received message with no delay. It can save energy; however, if all nodes have to rebroadcast a message, and the nodes that fail to demodulate a message have to remain active until it receives the message successfully from other nodes.

Meanwhile, many approaches are based on selective rebroadcasting. In Reference [5], they adjust a rebroadcast probability dynamically such that the more broadcast messages a node has received, the lower the probability of rebroadcasting it gets. In the dynamic probabilistic flooding algorithm based on neighbor information (DPFNI) protocol [6], a node also adjusts its rebroadcast probability such that the more siblings it has, the lower probability it gets, and the more children it has, the higher probability it gets. The rebroadcast probability is dynamically adjusted according to the change history of neighbor relationships after the specified times of flooding. Another approach builds a connected dominating set (CDS) such that flooding is completed if every node in a CDS rebroadcasts a message once [7]. In Reference [8], the protocol relies on slot scheduling to avoid collision, as well as the construction of a CDS. Finding the optimal CDS is known to be NP-complete [15]. Thus, in paper Reference [9], instead of finding a CDS, they try to assign a broadcast slot number incrementally to a node according to the distance from the node to a sink, and adjust the broadcast slot numbers for multiple nodes of the same distance to remove the possibility of collision. However, the change in topology causes the reconstruction of a CDS or the update of the random number table used for slot number assignment. In Reference [10], the authors use the opportunistic routing method, in which if a certain node happens to receive a broadcast message from one of its two-hop upstream nodes in the broadcast forwarding path and has higher connectivity than other nodes, it can forward the message earlier. A node can know whether or not its upstream node has received the message by using an acknowledgement (ACK) message. Meanwhile, in Reference [11], they propose a dynamic delegation-based efficient broadcast protocol (DDEB) in which if a node has any neighbor(s) who is uncovered (or failed to receive its broadcast message), it selects one of its covered neighbors based on link quality and asks it to cover the uncovered one. A broadcast node goes to sleep if all of its neighbors can be covered either directly or by one of its delegates. This approach tries to increase reliability by considering link quality. These approaches focus on reducing the number of rebroadcast nodes.

Some tree-based broadcasting methods have been proposed for energy saving and collision reduction. In Reference [12], they proposed a distributed heuristic algorithm to construct an energy-optimized tree under the delay bound. In Reference [13], they presented the delay-aware energy-optimized flooding (DEF) algorithm, in which each node reports the maximum delivery delay of its downstream nodes to the sink. The sink adjusts a flooding tree globally for energy efficiency under the delay bound. Another approach in this category is the reliable slotted broadcast protocol (RSBP) [14] that removes internal interference completely by allocating a distinct broadcast slot to each node except for leaf nodes. This approach optimizes energy consumption such that every node remains active only for its parent’s broadcast slot to receive a message and its own broadcast slot to rebroadcast it. However, it degrades reliability due to the complete removal of redundancy and has to reschedule the slots if the topology changes. Thus, these tree-based approaches may not be appropriate for dynamic WSNs.

In this paper, we improve reliability in message delivery and energy consumption by devising a slotted broadcasting method and applying it to the original SSMA broadcast (SSMAb) protocol [16] that was previously presented at a conference. We use the same protocol name, SSMAb. In SSMAb, one distinct broadcast sharable slot (BSS) is allocated to each tree level, and only the nodes at the same level compete to rebroadcast a message within the BSS, resulting in reduced competition. Every node can generate the schedule of BSSs based on tree levels, independently of the change in topology. Then, a node wakes up at the beginning of its parent’s BSS and goes to sleep immediately after receiving a message, and also wakes up at the beginning of its own BSS and goes to sleep immediately after rebroadcasting a message. In this way, a node can have multiple chances of receiving an identical message from different nodes at its parent level. However, collisions can still occur if two or more nodes broadcast a message simultaneously. To alleviate the possibility of collision, the protocol employs the concept of the slotted Aloha protocol [17]. A BSS is further divided into a number of broadcast slots (BSs), each BS being of a size sufficient to broadcast one message. Then, we present a BS scheduling algorithm with which a node can assign BSs to its children *on-the-fly* before rebroadcasting.

The SSMAb protocol was evaluated using the commercial simulator, QualNet 5.02, and was also experimented on in a testbed that consisted of one sink and 25 sensor nodes. It achieved a high reliability or high-packet delivery ratio comparable to the flooding scheme, and low-energy consumption comparable to the energy-optimized RSBP protocol against different degrees of interference and changes in topology. In addition, the delay bound was analyzed and compared with some broadcast protocols selected from each of the different categories explained above.

The rest of this paper is organized as follows. In Section 2, the preliminaries are given. Then, the proposed protocol is presented in Section 3, followed by Section 4 which analyzes and evaluates the protocol comparatively with some related approaches. Concluding remarks are given in Section 5.

## 2. Preliminaries

### 2.1. Network Model

A WSN consists of one *server* or one *sink node* and a number of *sensor nodes*. A sink is wall-powered while every sensor node is battery-powered. A node has a limited transmission range for energy saving as well as spectrum efficiency. Thus, the nodes can form a multi-hop tree originating from a sink. Two nodes that can communicate directly with each other are said to have a *link*. A link can be broken due to node mobility, node failure, battery depletion, or the intervention of some obstructions, thereby causing network topology to be changed dynamically. Especially, a link between a parent and its child is called a *tree-link.*

In tree topology, a server sends a command message to a specific node or a group of nodes whenever it needs. Basically, this can be achieved if every internal node rebroadcasts a received message after a server broadcasts the message. In this paper, we do not deal with the problem of minimizing the number of rebroadcasted nodes (see the optimized broadcast problem in Reference [18]).

### 2.2. Motivation

A simple flooding (*Flooding*) achieves high reliability in message delivery by allowing a node to have multiple chances of receiving or overhearing a broadcast message; however, it suffers from high-energy consumption. Let *AAT*(*X*) denote the *average active time* per each node with protocol *X*. In Flooding, each node remains active for half of the *broadcast period* (*BP*) on average since it goes to sleep immediately after rebroadcasting a received message. Furthermore, when *len*(*BS*) denotes the time length of *BS*, every node takes *len*(*BS*) approximately for rebroadcasting. Thus, we get:

(1) $$AAT\left(Flooding\right)=\frac{BP}{2}+len\left(BS\right)=\left(1+\frac{H}{2}\right)\times len\left(BS\right)$$

where BP=H×len(BS) when *H* is the depth of a tree in a tree-based protocol. Glossy [4] reduced *BP* by having a node rebroadcast a message immediately after it receives the message.

A reliable slotted broadcast protocol (RSBP) using a tree topology [14] optimizes energy consumption such that every internal node remains active only during two *BSs*, one to receive and another to rebroadcast a message, and a leaf node remains active for one *BS* to receive the message. Suppose that *r* denotes the ratio of leaf nodes in a given tree. Then, we get:

(2) AAT(RSBP)=(1−r)×2×len(BS)+r×len(BS)=(2−r)×len(BS)

However, the reliability is vulnerable to even the failure of a single link that may occur frequently in harsh industrial environments. For example, the failure of a node to receive a message can cause a chained failure such that all its descendants miss the message. In addition, a slot schedule has to be regenerated with the change of topology.

To tackle the weaknesses of Flooding and RSBP, we proposed a *slotted sense multiple access broadcast* (SSMAb) protocol [16] in which *multiple nodes* at the same tree level *compete* for broadcasting within the *broadcast sharable slot* (*BSS*) allocated to the tree level such that a node *senses a channel* and rebroadcasts a message only if the channel is idle. This approach still suffers from message collision and energy management within *BSS*. 

**Figure 1:**
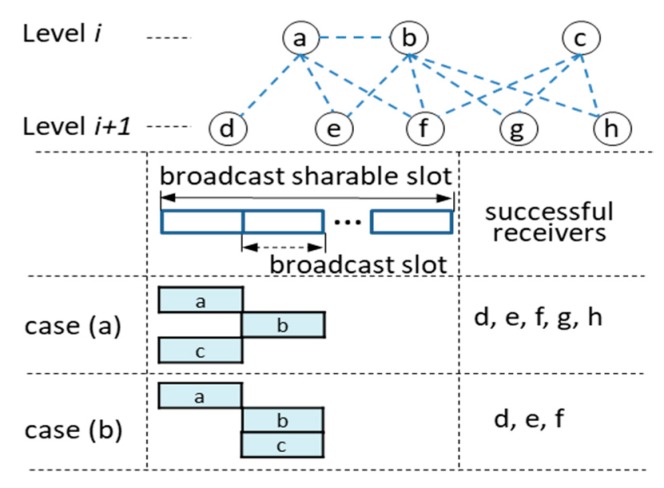
The effect on using the notion of the slotted Aloha within a broadcast sharable slot.

The original SSMAb can be improved by employing the notion of the slotted Aloha [17] since the broadcasting within one *BSS* is similar to data transmission in satellite networks except that a node knows its children and can sense the broadcast signal of its neighbors. Thus, as shown in Figure 1, a *BSS* is divided into multiple *BS*s and a node is allowed to start rebroadcasting only at the boundary of *BS*. However, the hidden nodes can still be problematic. Figure 1 illustrates two cases for the effect of a hidden node c: Case (a) that the broadcast of node *a* overlaps with that of node *c* and case (b) that the broadcast of node *b* overlaps with that of node *c*. In case (a), if node *f* fails to receive a message due to collision, fortunately it can be covered by node *b*. In case (b), if nodes *f*, *g,* and *h* fail to receive the message, nodes *g* and *h* cannot be covered by another node, *a*. To resolve this problem, we devised a *BS* scheduling algorithm in which *every node allocates a distinct BS to each of its children*. This implies that if nodes *b* and *c* have the same parent, they will be assigned different *BSs*, thereby preventing case (b). However, it is still possible that if two or more hidden nodes have different parents, they can be assigned the same *BS*. We do not provide a scheme to handle this problem in the *BS* scheduling algorithm. If that happens, the nodes that fail to receive a message due to collision have to rely on the possibility of being covered by another node as explained above.

Let *len(BSS*) be the time length of a *BSS*. Then, *AAT(SSMAb*) is given as follows:

(3) AAT(SSMAb)=max(len(BSS)2, len(BS))+[(1−r)×len(BS)+r×0]=max(0.5×len(BSS), len(BS))+(1−r)×len(BS)

where the first term indicates that each node remains active for at least one *BS* or half of the *BSS* on average since it goes to sleep as soon as it receives a message, and the second term indicates that each internal node remains active for one *BS* while every leaf node does not rebroadcast a message. Let len(BSS)=N×len(BS), Then, Equation (3) can be rewritten as follows:

(4) $$\begin{array}{cc} AAT\left(SSMAb\right) & =max\left(0.5N,\;1\right)\times len\left(BS\right)+\left(1-r\right)\times len\left(BS\right)\\ & =(max\left(0.5N,\;1)+1-r\right)\times len\left(BS\right) \end{array}$$

Note that *N* is reversely proportional to *H,* and thus, becomes smaller for taller trees. 

**Figure 2:**
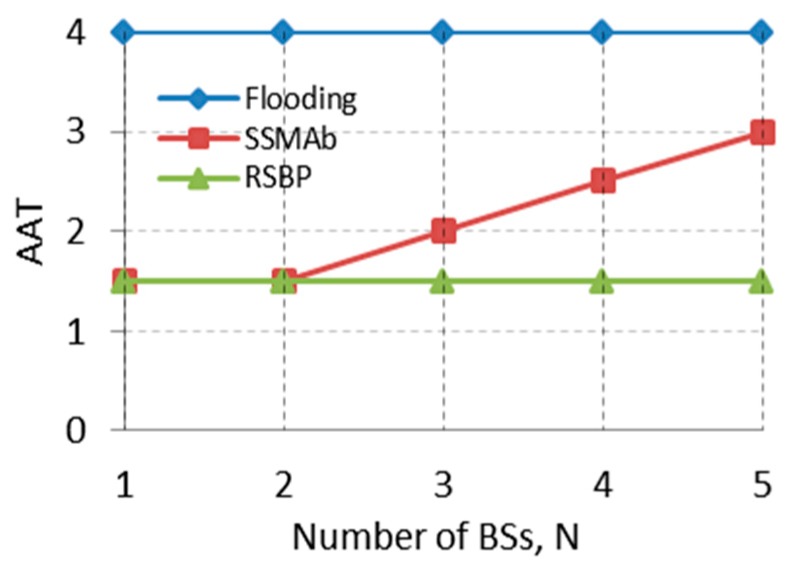
Comparison of average active time (*AAT*) per node for different approaches (assuming that len(*BS*) = 1, *H* = 6 and *r* ≈ 0.5).

Assuming that *r* ≈ 0.5 (a typical tree) and *H* = 6, Figure 2 compares three approaches where *AAT*(SSMAb) increases linearly with *N*. If N≤2, *AAT*(*SSMAb*) approaches *AAT*(*RSBP*) which shows optimal AAT, indicating that the improved SSMAb can manage energy well, in addition to improving reliability; however, *N* needs to be optimized.

## 3. SSMA Broadcast Protocol

A proposed protocol and its variations are described formally, starting with the explanation of protocol structure, and followed by analysis on key protocol parameters. Tree construction and time synchronization are not explained in this paper since they are detailed in the data collection protocol named SSMA [1]. Note that SSMAb is always used together with SSMA. One more thing to note is that tree construction and global time synchronization are performed one time at initialization time and maintained locally using control messages such as request to send (RTS), clear to send (CTS), and acknowledgement (ACK), which are used for reliable data transmission in SSMA. Thus, overhead is minimal.

### 3.1. Protocol Structure

**Figure 3:**
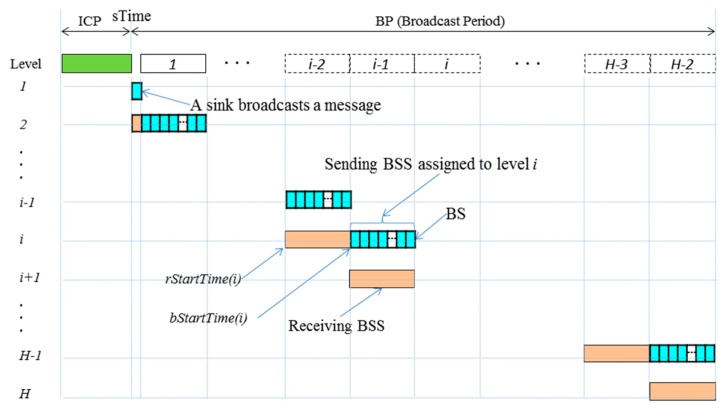
Protocol structure and the schedule of broadcast sharable slots (BSSs).

The protocol starts with *initial construction period* (ICP) and repeats *broadcast period* (BP) as shown in Figure 3. During ICP, time synchronization, tree construction, and BSS scheduling are performed. The BP starts with a globally synchronized time, *sTime*. During BP, broadcast is performed, starting with the message broadcast of a sink at tree level *one* and proceeding to next levels progressively. Given a tree of depth *H*, BP is divided into one BS and H-2 BSSs since nodes at level *H* do not have to rebroadcast a message, and each BSS is further divided into a number of BSs. During the (*i*−1)th BSS, every node at level *i* is in broadcast mode and goes to sleep immediately after rebroadcasting until the end of BP, while a node at level *i*+1 is in receiving mode and goes to sleep immediately after receiving until the start of its own sending BSS (ith BSS).

### 3.2. BSS Scheduling

As shown in Figure 3, all BSSs are of identical size. All nodes at the same level share one BSS. A receiving BSS allocated to level *i* completely overlaps with the sending BSS allocated to level *i*−1, since the nodes have to receive a broadcast message while their parents broadcast the message. Accordingly, a broadcast start time, *bStartTime*(*i*), a receiving start time, *rStartTime*(*i*), and the latest sleep time, *latestSleepTime*(*i*) of a node at level *i* are expressed as follows:

(5) $$bStartTime\left(i\right)=\{\begin{array}{cc} sTime, & i=1\left(sink\right)\\ sTime+len\left(BS\right)+\left(i-2\right)\times len\left(BSS\right), & 2\leq \;i\leq H-2 \end{array}$$

According to Equation (5), a sink starts broadcasting at *sTime* and nodes at level *i* ≥ 2 compete for broadcasting in (*i*−1)th *BSS* as illustrated in Figure 3.

(6) $$rStartTime\left(i\right)=\{\begin{array}{cc} sTime, & i=2\\ sTime+len\left(BS\right)+\left(i-3\right)\times len\left(BSS\right), & 3\leq \;i\leq H-3 \end{array}$$

All nodes wait for receiving a broadcast message within BSS allocated to their parent level. 

(7) latestSleepTime(i)=sTime+len(BS)+(i−1)×len(BSS), 2≤i≤H−1

Nodes always go to sleep at least at the end of their sending BSS while they can go to sleep early as soon as they finish rebroadcasting.

### 3.3. Broadcast Approaches

In this section, we describe some methods to reduce message collisions within BSS. Three progressively improved broadcast methods include a contention-based transmission (CBT), a slotted contention-based transmission (SCBT), and a slot-scheduled contention-based transmission (SSCBT).

#### 3.3.1. Contention-Based Transmission

In CBT, all the nodes at the same tree level compete for broadcasting. According to the IEEE 802.15.4 physical layer standard [19], a message transmission process takes seven steps: a sender (1) takes a random delay to avoid collision (*rdelay*); (2) transfers a message from micro controller unit (MCU) to a radio chip buffer (*t_mr_*); (3) turns the radio chip on (*t_turnon_*); (4) performs the clear channel assessment (CCA)(*t_CCA_*); (5) if the channel is idle, radio chip takes the physical layer processing delay (*t_ppd_*); (6) broadcasts a message (*t_tx_*); and upon receiving a message, (7) a receiver transfers a message from a radio chip to MCU (*t_rm_*). Therefore, to avoid collision, two neighboring nodes have to get the difference of *rdelay* by at least one *delayslot* that corresponds to the sum of *t_ppd_* and *t_CCA_*. This implies that one of two nodes never starts broadcasting before overhearing at least one bit transmitted earlier by another. With an integer contention window (*CW*), *rdelay* is given as follows:

(8) rdelay=random(0, CW)×delayslot

**Figure 4:**
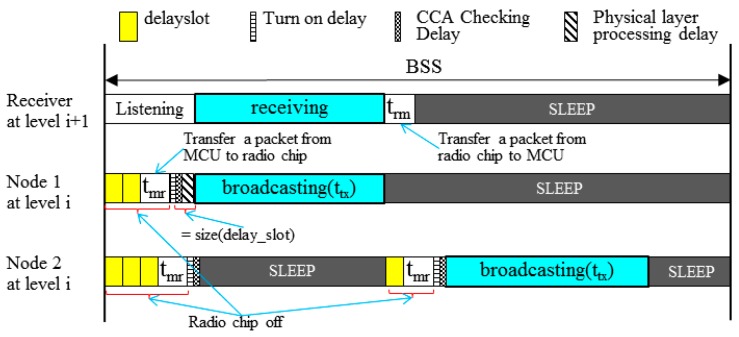
An example of a broadcast process between two neighboring nodes.

As in Figure 4, suppose that neighbors 1 and 2 generate random numbers, 2 and 3, respectively, from Equation (8). Then, node 1 takes the channel first, having node 2 delayed. The disadvantage of this approach is that since all nodes at the same level start channel competition simultaneously, some of them can generate an identical *rdelay*. This approach is similar to the way of broadcasting a message in the original SSMAb protocol [16], except that it uses the *rdelay* function.

#### 3.3.2. Slotted Contention-Based Transmission

A *slotted CBT* (SCBT) approach aims at alleviating the possibility of collision during message broadcasting by employing the slotted Aloha [17]. Thus, a BSS is further divided into N BSs as depicted in Figure 3, and can be expressed as a sequence of BSs:

$$BSS=\left(BS_{1},\;BS_{2},\;\ldots ,\;BS_{N}\right)$$

where $BS_{i}$ indicates the *i*th broadcast slot within *BSS* and N=⎣len(BSS)/len(BS)⎦. To alleviate competition, it is desirable to have nodes take different BSs if possible. Thus, every node, say *x*, chooses *BS* by using *startBS*(*x*) as follows:

(9) startBS(x)=random(1, N)

If either the number of nodes is much greater than the number of *BS*s, or the *BS* assignment by the random function is biased, multiple nodes can be assigned the same *BS*. Thus, nodes using the same *BS* contend for a channel using *rdelay* in Equation (8). If the current *BS* is the last one, a node that fails to acquire a channel has to rebroadcast a message. Otherwise, it is allowed to delay channel competition to the next *BS* only once.

This approach has a couple of shortcomings. First, some siblings can be assigned the same *BS*. Since they are located closely to each other, and thus are more likely to have connection to some nodes in common at one upper level, their simultaneous rebroadcasts can increase the possibility of collision. Second, if some hidden nodes connect to some nodes at one upper level in common, and are assigned the same *BS*, collision is inevitable regardless of the use of *rdelay*.

#### 3.3.3. Slot-Scheduled Contention-Based Transmission

**a. Slot scheduling**

In SSCBT, a node uses a BS scheduling algorithm that assigns a BS number to each of its children. Suppose that node *i* has *m* children expressed as $C\left(i\right)=\left(i_{1},\;i_{2},\ldots ,i_{m}\right)$ where $i_{j}$ indicates the *j^th^* child of node *i*. Given $BSS=\left(BS_{1},\;BS_{2},\;\ldots \;,\;BS_{N}\right)$, node *i* produces a *BS* schedule, *S*(*i*) for its children set C(i) as follows:

$$S\left(i\right)=\{\left(i_{j},\;BS_{k}\right)|j=1..m,\;k\;is\;selected\;by\;a\;BS\;scheduling\;Algorithm,\;1\leq k\leq N\}$$

The schedule *S*(*i*) implies that node $i_{j}$ is assigned $BS_{k}$. Node *i* includes *S*(*i*) in a message before rebroadcasting it so that each of its children can know its *BS* number.
**Algorithm 1** A broadcast slot (BS) scheduling algorithm//*N* = the number of *BS*s to be scheduled//*m* = the number of children//*BSN*(*i*) = the *BS* number assigned to node *i*If *i* is a sink then S(i)={(ij,BSk)|k=(j−1) mod N+1, 1≤j≤⎣m2⎦}
∪{(ij,BSk)| k=(j−(⎣m2⎦+1)+⎣N2⎦) mod N+1,⎣m2⎦<j≤m}If *i* is an intermediate node then $S\left(i\right)=\left\{\left(i_{j},BS_{k}\right)|\;k=\left(BSN\left(i\right)+j-2\right)\;mod\;N+1,\;1\leq j\leq m\right\}$


The BS scheduling algorithm works as follows. A node assigns a *BS* number to each of its children sequentially starting with its own *BS* number that was assigned by its parent. If a *BS* number to be assigned goes over the last one, it goes back to $BS_{1}$ and continues. If *N* is not smaller than *m*, every child will be assigned a unique *BS*. However, a sink that has no parent starts the *BS* scheduling with the first *BS*, *BS*_1_. Thus, a sink will assign only some *BS*s in front to its children, resulting in the unbalanced *BS* assignment. To avoid this situation, a sink generates a *BS* schedule such that it schedules the first half of children starting with the first *BS* and schedules the second half of children starting from the middle of *BS*s. For example, suppose that a sink, say 1, schedules $BSS=\left(BS_{1},BS_{2},BS_{3},BS_{4},BS_{5}\right)$ for $C\left(1\right)=\left(1_{1},1_{2},1_{3}\right)$. Then, it schedules the first half ($1_{1}$) of children starting with *BS_1_* and the second half ($1_{2},1_{3}$) starting with *BS*_3_. *S*(1) is given as follows:

$$S\left(1\right)=\left\{\left(1_{1},BS_{1}\right)\right\}\;\cup \;\left\{\left(1_{2},BS_{3}\right),\left(1_{3},BS_{4}\right)\right\}.$$

A detailed *BS* scheduling algorithm is given in Algorithm 1. The computational complexity of Algorithm 1 is O(*m*) where *m* ≤ *M* and *M* is the total number of nodes in the considered network.

**Figure 5:**
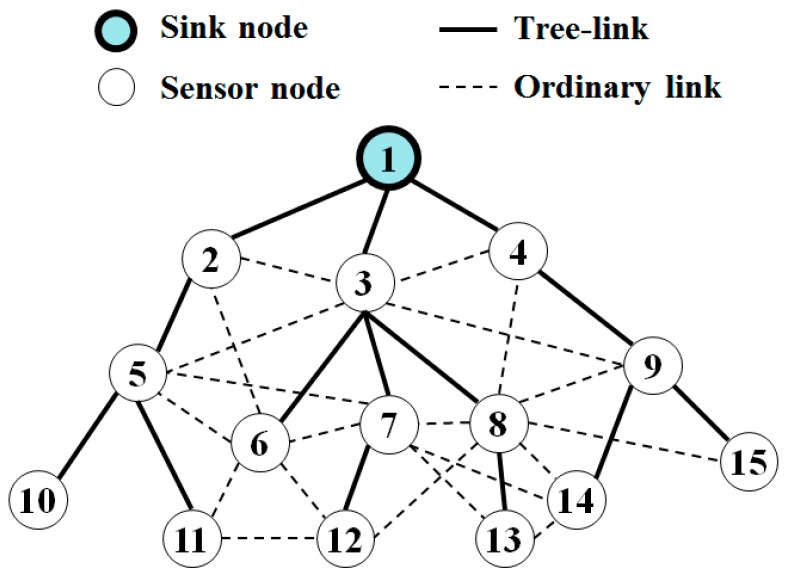
A wireless sensor network (WSN) topology of one sink and 14 nodes.

Let us generate a *BS* schedule for the tree in Figure 5. First, node 1 (a sink) has three children 2, 3, and 4. Then, the first half of nodes are {2} and the second half of nodes are {3, 4}. Thus, it assigns $BS_{1}$ to node 2 and $BS_{3}$ and $BS_{4}$ to nodes 3 and 4, respectively, resulting in S(1) = {(2, $BS_{1}$), (3, $BS_{3}$), (4, $BS_{4}$)}. Next, node 2 has one child 5 and starts slot assignment from its own $BS_{1}$. Thus, it assigns $BS_{1}$ to node 5, resulting in S(2) = {(5, $BS_{1}$)}. Node 3 starts slot assignment from its own $BS_{3}$ for its three children, resulting in S(3) = {(6, $BS_{3}$), (7, $BS_{4}$), (8, $BS_{1}$)}. Note that *BS_5_* going over the number of *BS*s is replaced by *BS_1_*. In this way, we get the *BS* schedules as shown in Table 1. Note that at tree level 2, hidden nodes 5 and 8 are assigned the same $BS_{1}$, and hidden nodes 7 and 9 are assigned the same $BS_{4}$ because they have different parents. If the hidden nodes 7 and 9 broadcast a message simultaneously, collision occurs at node 14; however, node 14 was already covered by node 8 at $BS_{1}$. 

In conclusion, SSCBT resolves the first shortcoming of SCBT since a node can assign different *BSs* to its children by using the *BS* scheduling algorithm. In addition, it alleviates the possibility of collision by hidden nodes since the closely located hidden nodes can be a sibling. Recall that the closely located hidden nodes at level *i* are more likely to have some nodes at level *i*+1 in common, but are more likely to have the same parent. For example, hidden nodes 6 and 8 connect to node 12 in common; however, they have the same parent.

**b. Message Broadcasting**

Upon receiving a message, if a node is not a leaf, it generates a *BS* schedule for its children *on-the-fly*, starting with its own *BS* number and then includes the *BS* schedule in the message before rebroadcasting. A node always saves a message that it overhears for the first time from nodes at its parent level. If a node fails to receive a message by the end of receiving *BSS*, it generates a *BS* schedule for its children using an arbitrary *BS* number and includes the schedule in the saved message before rebroadcasting. Thus, the protocol works well against a change of network topology. For message rebroadcasting, a node wakes up at its assigned *BS* and checks a channel after *rdelay*. If the channel is idle, it rebroadcasts the message immediately. If the current *BS* is the last one in *BSS*, it has no choice but to rebroadcast a message even though the channel is busy. Otherwise, it delays its rebroadcasting to the next *BS* only once and then rebroadcasts the message.

Furthermore, we can see that SSMAb enables a lot of parallelism. For example, referring to Figure 5, nodes 5 and 8 are assigned the same *BS*. However, they can rebroadcast the message simultaneously without causing collision at any receiver. Therefore, reliable broadcast can be achieved with a small number of *BS*s.

For mobile nodes, if a node does not receive a message during a certain BP, it wakes up one *BSS* earlier during next BP, assuming that the node has moved to one lower-level direction and remains active to overhear any message. If it overhears a message from multiple nodes, it chooses a node with the lowest tree level as its parent and resumes normal operation. This simple mechanism works well against mobile nodes. If SSMAb works with SSMA [1], the mobile node who has lost its parent can join its new parent during the data collection process by SSMA. However, since SSMAb is examined alone in this paper, we put *maintenance period* (*MP*) between two consecutive BPs. Thus, a mobile node can join its new parent by relying on the handshaking process of using *join request* and *join response* during MP.

### 3.4. Discussion on Key Protocol Parameter

#### 3.4.1. The Lower Bound of BS

In *SSCBT*, *len*(*BS*) should be optimized to reduce energy consumption. Since every message transmission goes through seven steps given in Section 3.3.1 within *BS*, the lower bound of *len*(*BS*) is given as follows:

(10) $$len\left(BS\right)\geq max\left(rdelay\right)+t_{mr}+t_{turnon}+t_{CCA}+t_{ppd}+t_{tx}+t_{rm}$$

#### 3.4.2. The Lower Bound of BSS

The length of *BSS* should be able to handle the largest possible competing nodes, while it should be optimized to decrease message broadcast delay. Then, the lower bound of *len*(*BSS*) is given as follows:

(11) len(BSS)≥len(BS)×N

The integer number *N* can be determined by estimating the maximum number of competing nodes. Let *nCNodes* and *nNbrs* be the number of competing nodes and the number of neighbors for a given node, respectively. With SSMAb, *nCNodes* is approximately the same as one third of *nNbrs* since a node has its neighbors over three tree levels. Thus, *nCNodes* is given as follows:

(12) nCNodes ≈ ⎡nNbrs3⎤+1

**Figure 6:**
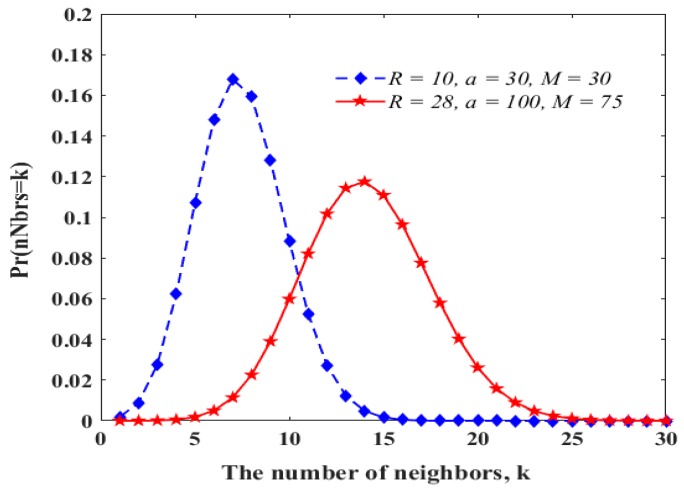
Distribution of node degrees.

**Figure 7:**
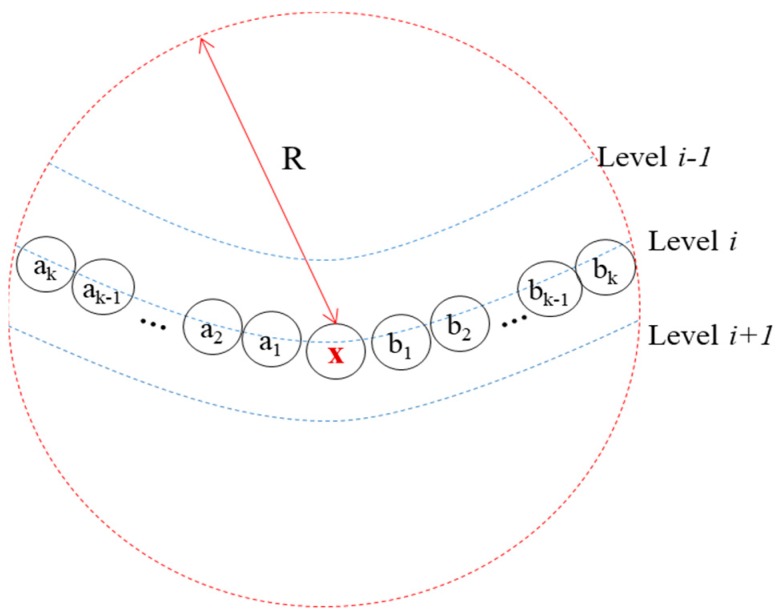
The maximum number of competing nodes at any tree level.

From the probability mass function in Reference [20], the distribution of *Pr*(*nNbrs* = *k*) is shown in Figure 6, where *R*, *a*, and *M* indicate the transmission range, the width or the length of network dimension, and the number of nodes, respectively. However, take a look at Figure 7, where node *x* at level *i* has 2 × *k* neighbors, corresponding to the maximum number of neighbors that a node can have at the same level, and the big dotted circle indicates the coverage of node *x*. When node *x* gets the channel, all its neighbors at the same level are blocked, showing the worst-case blocking. Note that if any node (say $a_{1}$) other than node *x* gets one *BS*, one ($b_{k}$) of the other nodes can use the same *BS* simultaneously. Thus, after node *x* broadcasts a message, two nodes can broadcast a message within the same *BS* in parallel. This continues until all nodes finish transmission. Thus, we get *N* from *nCNodes* in Equation (12) approximately as follows:

(13) N≈ ⎡12×nCNodes⎤

We can get the maximum possible number of neighbors from Figure 6. Thus, when *R* = 10 m, a = 30 m, and *M* = 30, *nNbrs* ≈ 16, and *nCNodes* ≈ 7 from Equation (12), and *N* ≈ 4 from Equation (13). When *R* = 28 m, a = 100 m, and *M* = 75, *nNbrs* ≈ 25, *nCNodes* ≈ 10, and *N* ≈ 5.

#### 3.4.3. The Lower Bound of BP

Since every level except for level one takes one *BSS* and the nodes at the highest level do not have to rebroadcast the message, the lower bound of *BP* for a tree of depth *H* is given as follows:

(14) BP≥len(BS)+(H−2)×len(BSS)

**Figure 8:**
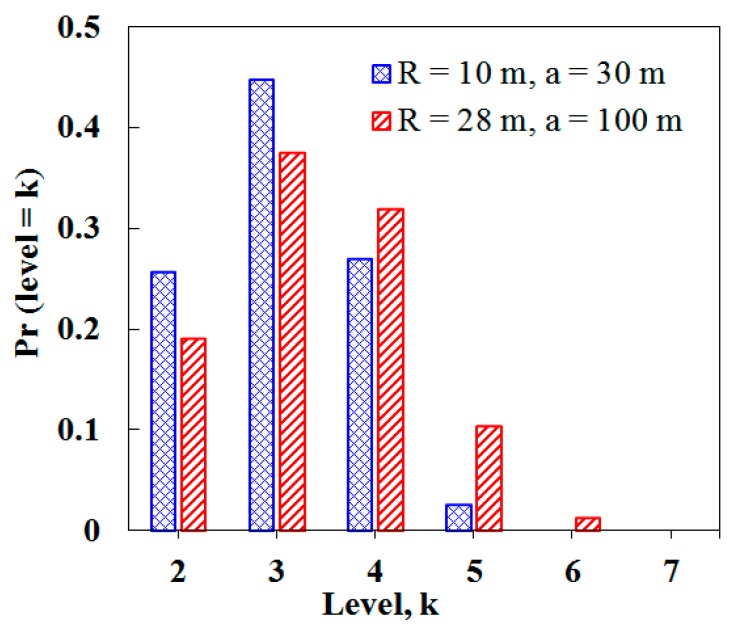
Probability distribution of tree levels.

We need to estimate *H* that corresponds to the maximum level. From the cumulative distribution function in Reference [21], the distribution of *Pr*(*level* = *k*) is shown in Figure 8 where *R* and a indicate the transmission range and the width and length of network dimension, respectively.

From Figure 8, when *R* = 10 m and α = 30 m, *H* = *k* ≈ 5 (i.e., *Pr*(*level* = 5) ≈ 0.0254), and when *R* = 28 m and α = 100 m, *H* *= k* ≈ 6 (i.e., *Pr*(*level* = 6) ≈ 0.012).

### 3.5. SSMAb Characteristics

Table 2 compares the proposed SSMAb and three approaches selected from each of the broadcast protocol categories qualitatively. Flooding and DPFNI [6] have merits in all features except for energy consumption. In RSBP [14], every node manages energy optimally in that it remains active only at sending and receiving broadcast slots, does not allow overhearing, and allows a node to rebroadcast a message only if it is not a leaf node. The SSMAb protocol has good characteristics in reliability of message delivery comparable to Flooding and energy management comparable to RSBP. It also can have lower end-to-end delay than RSBP since it allows concurrency in broadcasting. Furthermore, it can respond well against changes in topology since the *BSS* schedule is topology-independent.

## 4. Performance Evaluation

### 4.1. Analysis of End-to-End Delay

We calculated the worst-case end-to-end delay (*E2ED*) that corresponded to BP for the four broadcast protocols: Glossy [4], DPFNI [6], RSBP [14], and SSMAb.

In Glossy [4], the time to send a packet, $T_{tx}$, is given as follows:

(15) $$T_{tx}=T_{sw}+T_{cal}+T_{pr}+T_{f}+T_{l}+T_{m}=375.5\left(\mu s\right)+T_{m}$$

where, $T_{sw,}T_{cal}$, $T_{pr}$, $T_{f}$, $T_{l}$, $T_{m}$ are software delay, calibration time, preamble time, SFD (start of frame delimiter) time, length in time, and MAC protocol data unit (MPDU) time, respectively. Note that in Equation (15), *T_m_* is the time to send payload of size *p*. Therefore, in the 802.15.4 physical layer standard, the bandwidth was 250 kbps and each packet took an additional 6 bytes for synchronization header, PHY header, and MAC header and footer. Thus, the *E2ED* of Glossy is given as follows:

(16) $$E2ED\left(Glossy\right)=H\times T_{tx}=H\times \left(0.3755+\left(6+p\right)\times 0.032\right)\left(ms\right)$$

In DPFNI [6], it uses the back-off time before retransmission, and its *E2ED* is given as follows:

(17) $$\begin{array}{ll} E2ED\left(DPFNI\right) & =H\times T_{B}\\ & =H\times \left(T_{Backoff}+T_{ppd}+T_{tx})=H\times (3.192+\left(6+p\right)\times 0.032\right)\left(ms\right) \end{array}$$

where $T_{B}$ is a broadcast time as the sum of the back-off time ($T_{Backoff}$), the physical layer processing delay ($T_{ppd}$), and transmission time ($T_{tx}$), and $T_{Backoff}$ in the paper is not bigger than 3 ms.

In RSBP [14], only non-leaf nodes rebroadcast a message and one distinct *BS* is allocated to each of the non-leaf nodes. Let *nBNodes* denote the number of broadcast nodes. Then, the *E2ED* of RSBP can be expressed as follows:

(18) E2ED(RSBP)=nBNodes×len(BS)=nBNodes ×(0.192+(6+p)×0.032)(ms)

where $len\left(BS\right)=t_{mr}+t_{turnon}+t_{rm}+t_{ppd}+t_{tx}$ and $t_{mr},\;t_{turnon}$ and $t_{rm}$ are assumed to be zero in simulation.

In SSMAb, rewriting Equation (10), we get *len*(*BS*) as follows:

(19) len(BS)=((CW+1)×0.32+(6+p)×0.032) (ms)

where max(*rdelay*) = CW x *delay_slot*, *delay_slot* = 20 symbols delay, t_CCA_ = 8 symbols delay, $t_{ppd}$ = 12 symbols delay, and one symbol delay = 0.016 ms. Rewriting Equation (14) with Equations (11) and (19), we get:

(20) E2ED(SSMAb)≥((H−2)×N+1)×len(BS)

**Figure 9:**
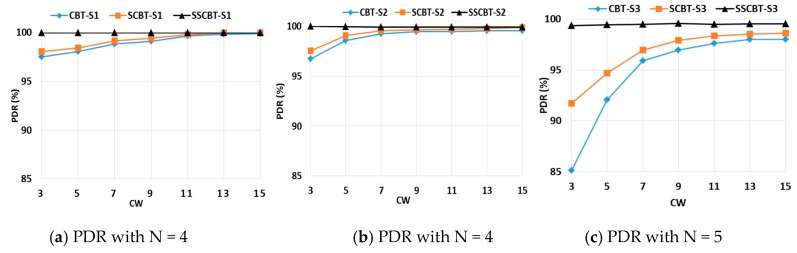
Packet delivery ratios for the three broadcast methods with varying CW.

From Equations (16)–(18) and (20), the *E2ED*s for the four broadcast protocols are compared in Table 3. The *CW* value for SSMAb is set to three since it is good enough for both 30 nodes and 75 nodes according to simulation results in Figure 9 later. The *E2ED*s of SSMAb for 30 nodes and 75 nodes are given 60.7 ms and 98.1 ms, respectively. Furthermore, it shows good *E2ED* comparable to RSBP for 30 nodes due to low tree depth. Glossy achieves the smallest E2ED of all since it does not use random delay before rebroadcasting. We believe that about 100 ms is sufficiently small to satisfy the time bound of command delivery in industrial monitoring and control applications.

### 4.2. Simulation

#### 4.2.1. Simulation Setup

A sink broadcasts one message every BP with the MAC payload fixed to 100 bytes until the simulation ends. The key simulation parameters and values for static scenarios S1, S2, and S3 are given in Table 4, and those for dynamic scenarios, S2M and S3M, are given in Table 5. Scenario S1 was chosen to reflect a WSN at a long rectangular zone such as an underground tunnel and a factory assembly line. In dynamic scenarios, some nodes in percentage are allowed to move using the random waypoint model with the minimum speed of 1.0 m per second, the maximum speed of 1.5 m per second, and the pause time of 5 s. Considering the short transmission range of 10 m, the total simulation time was set to 200 s, sufficient to make the change of topology to a considerable extent. To create a fading effect, we employed the Ricean fading model with driving parameter *K*, which is defined as the ratio of the receiving power in the direct path (line of sight) to that in other paths. We changed the value of *K* to adjust the strength of the fading effect.

Packet delivery ratio (PDR) is the ratio of the number of nodes that received a message to the total number of nodes. Packet processing load (PPL) is the number of messages that each node received or overhears and also transmits on average per each broadcast message. Average energy consumption (AEC) is the average amount of energy consumption per node during the total simulation time. 

#### 4.2.2. Discussion of Simulation Results

**a. Comparing Three Broadcast Methods**

With scenarios S1, S2, and S3, we compared PDRs for the three broadcast methods, CBT, SCBT, and SSCBT. From the analysis in Section 3.4.2, *N*, the number of BSs, was set to 4 for S2 and 5 for S3.

Figure 9 shows that the PDRs of the three approaches depended on how well they could handle competing nodes. The SSCBT achieved almost 100% PDR regardless of the size of CW, while the others showed decreasing PDRs as CW decreased. It was obvious that the PDR of SCBT approached that of SSCBT as CW increased. We can see that SSCBT was not sensitive to the type of scenario or the CW size since it used a BS scheduling. According to Equations (8) and (10), the smallest possible size of CW has to be selected to reduce *len*(*BS*). From this study, CW was set to three or less for the rest of the simulation.

**b. Optimal Number of BSs with SSCBT**

**Figure 10:**
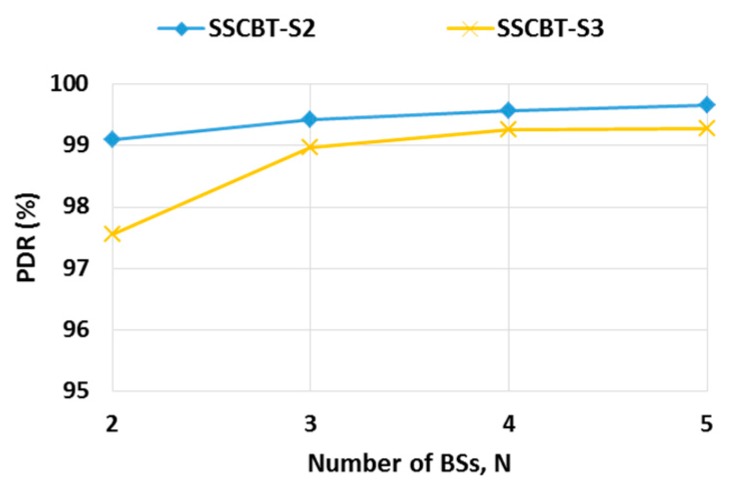
The optimal number of BSs with CW fixed to three.

To verify the *N* values analyzed in Section 4.2, we conducted simulations with S2 and S3 in Table 4. We examined SSCBT with varying *N* from two through five with CW fixed to three from Section 4.2.2(a). Referring to Figure 10, the increase of PDR almost stopped at *N* = 4 after reaching over 99% for both S2 and S3. This implies that SSCBT allowed the high degree of parallelism since *N* is much less than the number of nodes at the same level. *N* = 4 accords very well with the analytical value computed by Equation (13) (even though *N* = 5 for S3 analytically).

**c. Comparison of Protocols with Varying Fading Effects**

**Figure 11:**
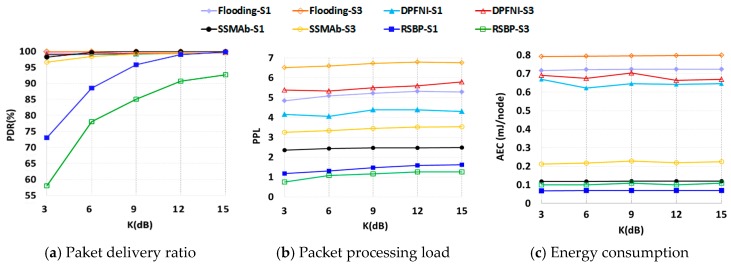
Comparison of different protocols with varying K (*N* = 4).

In Figure 11a, it is shown that Flooding and DPFNI are very reliable even with *K* = 3 (very high fading effect). The SSMAb protocol shows a high PDR comparable to Flooding with S1. This is because with SSMAb, a node has multiple chances of receiving a message even though it broadcasts and receives or overhears the number of messages less than Flooding and DPFNI, as shown in Figure 11b. However, it achieves slightly lower PDR overall with S3 if *K* is less than or equal to six.

Another important feature is that it can achieve low-energy consumption comparable to that of RSBP, which shows optimal energy saving as shown in Figure 11c. This is because in SSMAb, a node remains active during half of the receiving BSS on average to receive a message and during the allocated BS for rebroadcasting only if it has a child (see Section 2.2). This is the reason that SSMAb achieves almost the same energy consumption as RSBP. Furthermore, it is shown that AEC is not affected by the fading channel factor *K*. Note that every node rebroadcasts a message at most once in all protocols since a broadcasting node does not check the message reception of a receiver. Thus, the link breakages affect energy consumption a little. Furthermore, in RSBP and SSMAb, only an internal node rebroadcasts a message. Thus, the change in link condition may increase or decrease the number of internal nodes; however, the ratio of internal nodes will remain almost same on average. Thus, it is obvious that the decrease of *K* does not increase AEC. Similarly, in DPFNI, a node rebroadcasts a message depending on its broadcast probability which is affected by its connectivity as explained in the Introduction. Thus, it still does not increase AEC noticeably, ether.

**d. Comparison of Different Protocols with Node Mobility**

**Figure 12:**
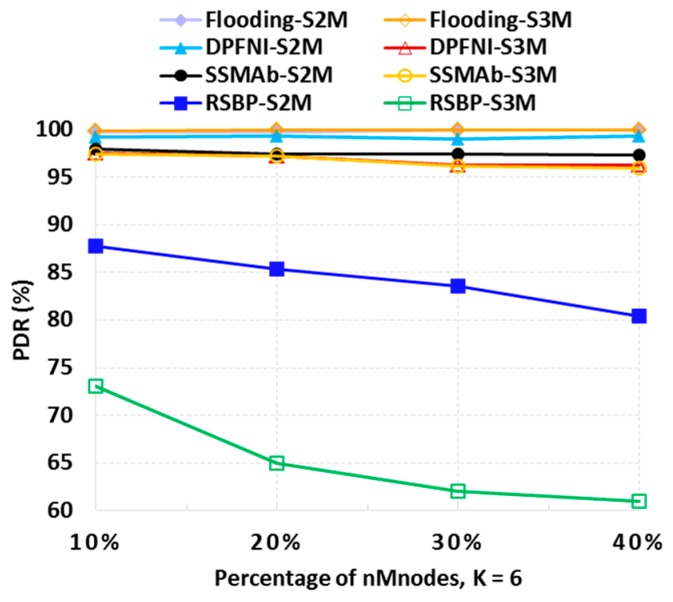
Packet delivery ratio according to the percentage of mobile nodes (*K* = 6).

In Figure 12, the PDRs of different protocols were examined by changing the number of mobile nodes (*nMNodes*) from 10% to 40% with *K* fixed to six. It was shown that the PDR of Flooding was not affected by the increase of *nMNodes*. The DPFNI protocol was also considerably reliable against node mobility. However, note that SSMAb also achieved high reliability comparable to DPFNI overall against node mobility, while it consumed low energy, as shown in Figure 11c. As expected, it is proven that RSBP does not respond well to node mobility.

### 4.3. Experiment

#### 4.3.1. Experiment Setup

**Figure 13:**
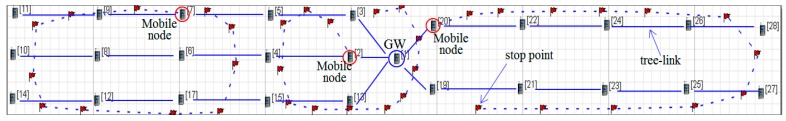
Tree network topology with the traces of mobile nodes for both simulation and experiment (H = 6, average speed of 1 m per second, and pause time of 5 s at each stop).

For the experiments, we used the *usens* mote developed in our lab, running ContikiOS 2.6. It was equipped with Texas Instrument’s CC2630 which integrates the ultra-low-power ARM CorTex M3 and CC2420 radio chip (that conforms to a 250 kbps 2.4 GHz IEEE 802.15.4 wireless transceiver). Micro controller unit has 20 KB of RAM and 128 KB of flash memory. The current draw, excluding the radio, was 1.45 mA in active mode and 1.2 μA in sleep mode. The CC2630 consumed 5.9 mA in receiving or listening mode, and 9.1 mA when transmitting at level +5 dBm [22]. One gateway (GW) and twenty-five sensor nodes were artificially deployed along a corridor in the building of the computer engineering department in the University of Ulsan, as shown in Figure 13, with an equal distance of about 7 m between parent and child. The GW is located at the middle. In the experiments, 1 to 3 selected nodes were moved at an average speed of 1 m per second along the traces, pausing at each stop point for 5 s. The other key experimental parameters and values are shown in Table 6.

#### 4.3.2. Determination of Protocol Parameter Values for Experiment

When the MAC frame was forwarded from MCU to the radio chip in CC2630, 6 bytes were added to the MAC frame for transmission preamble and frame delimiter when the message was transmitted over the medium to the destination, in order to be compatible with the IEEE 802.15.4 PHY standard [19]. In our implementation, the address information field in MPDU was removed since a command message was broadcasted; other elements were kept in order to keep compatible with the IEEE 802.15.4 standard. We assumed that the MAC payload *p* was 100 bytes long. On the sender side, a MAC frame of (3+*p*) bytes, i.e., size(MHR) + *p*, was transferred from MCU into the radio chip, while on the receiver side, a MAC frame of (5+*p*) bytes, i.e., size(MHR) + size(MFR) + *p*, was transferred from the radio chip into MCU (MHR: MAC header, MFR: MAC footer). The total size of a frame when it is transmitted over the medium is size(SHR) + size(PHR) + size(MHR) *+ p* + size(MFR) (SHR: synchronization header, PHR: Physical layer header).

The message transfer times, $t_{mr}$ and $t_{rm}$, were determined by the speed of serial peripheral interface (SPI), whereas transmit time $t_{tx}$ was determined by the transmission speed of the radio chip. The typical Internal Digitally Controlled Oscillator (*DCOCLK*) clock frequency of the ARM CorTex M3 with the default configuration of the ContikiOS was 3.2 MHz. Therefore, the maximum SPI rate was half of the *DCOCLK* clock frequency (=1.6 MHz). Based on our ContikiOS interface experiment, the SPI interface on *usens* achieved a rate of 4 Mbps (≈0.25 μs per bit). Additionally, t_turnon_ ≈ 100 µs, t_CCA_ = 128 µs, and *t_ppd_* = 192 µs. Based on the size of the frame format at each layer, the processing delay, and the SPI speed, the practical values of $t_{mr}$, $t_{rm}$, and $t_{tx}$ were computed as follows (FCH: Frame control header).

$$\begin{array}{l} t_{mr}=(size(MHR)+p)\times 8\times 0.25\mu s=(3+p)\times 2\times 10^{-3}(ms)\\ t_{rm}=(size(MHR+MFR)+p)\times 8\times 0.25\mu s=(5+p)\times 2\times 10^{-3}(ms)\\ t_{tx}=(size(MHR+SHR+PHR+FCH)+p)\times 8\times 4\mu s=(11+p)\times 32\times 10^{-3}(ms) \end{array}$$

By applying these values to Equation (10), we get *len*(*BS*) as follows:

(21) $$len\left(BS\right)=CW\times 0.32+0.788+36\times p\times 10^{-3}\left(\mathrm{ms}\right)$$

Using *len*(*BS*) and the parameter values given in Table 6, we obtained the following values from Equations (10), (11), and (14): len(BS)≈5.35 ms, len(BSS)≈16.1 ms, len(BP)≈64.4 ms.

As for the values of *H* and *N*, *H* depends on the dimension of a target network and the transmission range of a node, and *N* depends on the number of neighbors that varies according to the node density of a target network and the transmission range of a node. In case of the tree topology in Figure 13 that is deployed artificially where *H* is six, *N* is set to three since the number of competing nodes at the same level is equal to three. 

Consider a tree of a randomly deployed network as in scenario S2 in Table 4, where *H* = 5. In this network, *N* is set to 4 (BSs) by analysis and simulation. However, in real situations, *H* can be used as any value greater than or equal to 5, the real depth of a tree. For example, suppose that *H* is set to six conservatively. In fact, the use of the bigger *H* value does not increase end-to-end delay and energy consumption according to the operation of SSMAb. Meanwhile, *N* can be set roughly by considering the connectivity of the network since the sensitivity of *N* is extremely low due to the parallelism in broadcasting as shown in Figure 10. If *N* is three or bigger, PDR is over 99% for both S2 and S3. Even though *N* is set to four conservatively, it will increase one *BS* in every *BSS*. This will increase only (H-2) *BS*s (=21.4 ms if *H* = 6) since the network uses (*H*-2) *BSS*s. It will also increase energy consumption a little since a node has to remain active for *N*/2 BSs on average to receive a message, thus increasing the active time by 1/2 *BS* (=2.33 ms) since *len*(*BS*) = 4.67 ms from Table 3.

In conclusion, *H* can be set to a proper value considering dimension and transmission range. One way to use an optimal value of *H* is that every node transmits its level at tree construction time once. Then, *H* can be set to the highest level plus one by considering the possible change of topology. *N* can be set to three as a default value, and *N* = 4 will be big enough for any network topology.

#### 4.3.3. Discussion of Experiment Results

**Figure 14:**
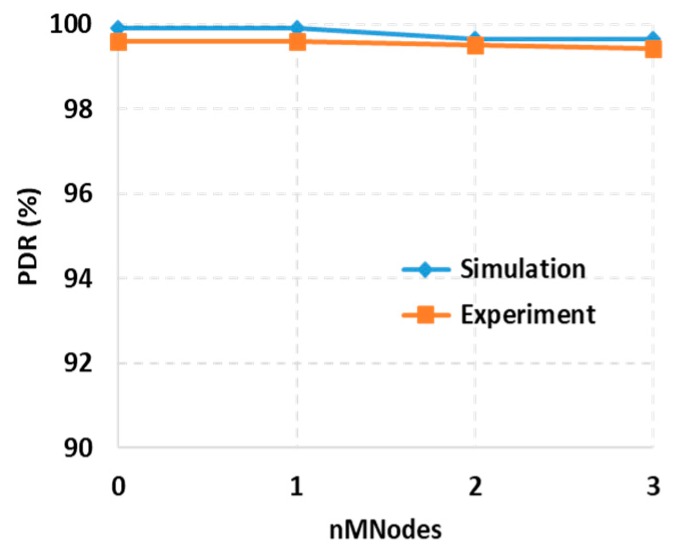
Comparison of packet delivery ratio for experiment and simulation (*K* = 6 in simulations).

Figure 14 compares the PDRs of SSMAb for simulation and experiment where PDR in the testbed was slightly lower than that in the simulations overall. The wireless communication in a real environment is affected by various factors such as the quality of the antenna, the correctness of CCA checking, and external interference from other wireless networks. Thus, *K* was set to 6 in the simulations. The PDR was always higher than 99.5% in the experiments. Furthermore, it was clearly seen that with the increase in the number of mobile nodes, the PDR of SSMAb was well sustained.

## 5. Concluding Remarks

The proposed broadcast protocol, SSMAb, cannot only deliver a message in a reliable manner with good delay bound, but also in an energy-efficient manner. It works well against the change of topology in dynamic wireless sensor networks and high internal and external interference because it uses a topology-independent slot schedule and allows a node to have multiple chances of receiving an identical message. It also manages energy efficiently by using broadcast sharable slots and performing a slot scheduled broadcast. According to simulations and experiments, it shows high energy efficiency comparable to RSBP, which is optimal in energy consumption while it achieves a high packet delivery rate comparable to the flooding approach that shows the best reliability.

## Figures and Tables

**Table 1:** A broadcast slot (BS) schedule for the tree in Figure 5.

Tree Level	BS Schedule	Broadcast Slots in BSS			
		$BS_{1}$	$BS_{2}$	$BS_{3}$	$BS_{4}$
1	S(1)	2		3	4
2	S(2)	5			
	S(3)	8		6	7
	S(4)				9
3	S(5)	10	11		
	S(7)				12
	S(8)	13			
	S(9)	15			14
X: node X does not rebroadcast a message since it does not have a child					

**Table 2:** Comparison of the characteristics of SSMAb and other approaches.

Features	Flooding	DPFNI	RSBP	SSMAb
Reliability of message delivery	Multiple chances of receiving a message	Multiple chances of receiving with probability	A single chance of receiving with no collision	Multiple chances of receiving with reduced collision
	Very high	High	Mid	High
Delay in message delivery	Random delay	Random delay	Time bound by slot schedule	Time bound by sharable slot schedule
	Low	Low	High	High
Concurrency of broadcast	Free broadcast after random delay	Free broadcast after random delay	Scheduled broadcast	Scheduled concurrency at the same tree level
	High	High	Zero	Mid
Energy consumption	Rebroadcasting by all nodes and long active time	Rebroadcasting with probability and long active time	Rebroadcasting by internal nodes only and managed active time	Rebroadcasting by internal nodes only and managed active time
	High	High	Optimal	Low
Responsiveness to dynamic topology	No topology	Topology-dependent probability	Tree topology and topology-dependent	Tree topology and topology-independent
	High	Mid–High	Low	Mid–High
Scheduling overhead	No scheduling	No scheduling	Centralized scheduling	Distributed scheduling
	No	No	High	Almost negligible

**Table 3:** Comparisons on the lower bound of E2ED.

Parameters	SSMAb (CW = 3)	RSBP	GLOSSY	DPFNI	SSMAb (CW = 3)	RSBP	GLOSSY	DPFNI
*Len(BS)* (ms)	4.67	3.58	n/a	n/a	4.67	3.58	n/a	n/a
N	4	n/a	n/a	n/a	5	n/a	n/a	n/a
Depth H	5	n/s	5	5	6	n/s	6	6
nBNodes	n/s	15	n/a	n/a	n/a	26	n/a	n/a
E2ED (ms)	60.7	53.7	18.9	32.9	98.1	93.1	22.6	39.5
Common	Dimension = 30 × 30 (m^2^), #Nodes = 30, R = 10 m, p = 100 bytes				Dimension = 100 × 100 (m^2^), #Nodes = 75, R = 28 m, p = 100 bytes			

**Table 4:** Three static network scenarios and parameter values.

S1	S2	S3
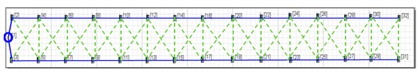	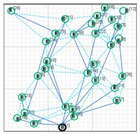	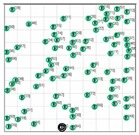
Dimension: 10 × 90 (m^2^)	30 × 30 (m^2^)	100 × 100 (m^2^)
Number of nodes: 1 sink + 30 nodes	1 sink + 30 nodes	1 sink + 75 nodes
Transmission range: 10 m (−29 dBm)	10 m (−29 dBm)	28 m (−24 dBm)
Node distribution: Artificial	Random	Random

**Table 5:** Two dynamic network scenarios corresponding to S2 and S3 in Table 4.

Parameters	S2M	S3M
Node distribution with the trace of node movement	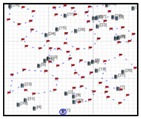	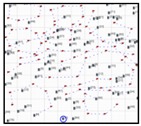
Mobility model	Random waypoint	
% of mobile nodes (*nMNodes*)	10%, 20%, 30%, 40%	
Average speed (mps)	[1.0, 1.5]	
Pause time (seconds)	5	

**Table 6:** Key experimental parameters and values for experiment.

Parameters	Values
Number of nodes	25 nodes and 1 sink
*N*	3
*CW*	3
Transmission power	−12 dBm (≈10 m)
Number of mobile nodes	1 to 3
Dimension	About 2.5 m × 70 m
Payload, *p*	100 bytes
